# Enhancing Ecoefficiency in Shrimp Farming through Interconnected Ponds

**DOI:** 10.1155/2015/873748

**Published:** 2015-10-07

**Authors:** Ramón Héctor Barraza-Guardado, José Alfredo Arreola-Lizárraga, Anselmo Miranda-Baeza, Manuel Juárez-García, Antonio Juvera-Hoyos, Ramón Casillas-Hernández

**Affiliations:** ^1^Programa Doctorado en Ciencias en Biotecnología, Instituto Tecnológico de Sonora (ITSON), Bulevar 5 de Febrero 818 Sur, 85000 Ciudad Obregón, SON, Mexico; ^2^Departamento de Investigaciones Científicas y Tecnológicas de la Universidad de Sonora (DICTUS), Bulevar Colosio s/n, Edificio 7J, 83000 Hermosillo, SON, Mexico; ^3^Centro de Investigaciones Biológicas del Noroeste S.C., 85454 Guaymas, SON, Mexico; ^4^Laboratorio de Tecnologías de Cultivo de Organismos Acuáticos, Universidad Estatal de Sonora (UES), 85800 Navojoa, SON, Mexico; ^5^Instituto Tecnológico Superior Zacatecas Norte, 98400 Río Grande, ZAC, Mexico; ^6^Acuícola Polo, S.A. de C.V., Bulevar Lázaro Cárdenas No. 940, Colonia Las Ladrilleras, 83127 Hermosillo, SON, Mexico

## Abstract

The future development of shrimp farming needs to improve its ecoefficiency. The purpose of this study was to evaluate water quality, flows, and nitrogen balance and production parameters on a farm with interconnected pond design to improve the efficiency of the semi-intensive culture of *Litopenaeus vannamei* ponds. The study was conducted in 21 commercial culture ponds during 180 days at densities of 30–35 ind m^−2^ and daily water exchange <2%. Our study provides evidence that by interconnecting ponds nutrient recycling is favored by promoting the growth of primary producers of the pond as chlorophyll *a*. Based on the mass balance and flow of nutrients this culture system reduces the flow of solid, particulate organic matter, and nitrogen compounds to the environment and significantly increases the efficiency of water (5 to 6.5 m^3^ kg^−1^ cycle^−1^), when compared with traditional culture systems. With this culture system it is possible to recover up to 34% of the total nitrogen entering the system, with production in excess of 4,000 kg ha^−1^ shrimp. We believe that the production system with interconnected ponds is a technically feasible model to improve ecoefficiency production of shrimp farming.

## 1. Introduction

The future development of shrimp farming requires innovative and responsible practices to improve their operating efficiency and help prevent environmental degradation of coastal ecosystems [1]. Some proposals include the use of mangroves as biofilters of crop effluents [2], performing polycultures with seaweed and shellfish [3, 4], the use of microbial mats in ponds [5], farming systems with low water exchange [6], and strategies for cleaner power [7]. Exchange or recycling of the water in the ponds serves to keep the water variables in conditions suitable for the growth and development of shrimp. However, rates of over 16% water exchange increase operating costs, such as the amount of fuel used, as well as increasing the quantity of pollutant inputs [8]. Semi-intensive shrimp farming of northwestern Mexico can have water exchange rates greater than 25% water [9], but mass mortality events in 2010, 2011, 2012, and 2013 due to the presence of diseases recommend reducing water turnover rates [10]. In aquaculture systems with low water turnover rates autotrophic, chemoautotrophic, and phototrophic processes have been studied, and a rapid increase in organic matter has been observed, which can serve as a substrate for the development of heterotrophic bacteria; on the other hand nitrogen compounds are remineralized by nitrifying bacteria and are consumed by microalgae. These processes allow for potentially polluting compounds to enter the food chain [11–14]. High turnover rate allows for some water quality variables to be well regulated in terms of water quality; it nevertheless represents a massive waste of potentially useful nutrients and organic matter. Martínez-Córdova et al. [15] demonstrated experimentally that it is possible to reuse the effluent of semi-intensive ponds to grow bivalves, benthic diatoms, and whiteleg shrimp (*Litopenaeus vannamei*) in a multitrophic system. Nevertheless, this practice requires validation for use on a commercial scale because the effects on water quality and productive performance of shrimp, as well as N recycling and discharge, are unknown. It is widely documented that only between 18 and 27% of N entering the ponds is converted into shrimp biomass; the rest is discharged into the environment [16–19]. Most of N entering the ponds exits through effluents during water changes. Water exchange in addition to influencing discharge potentially releases harmful components for the environment, representing huge volumes of water masses that move annually between coastal water bodies and fish farms. In the northwest of Mexico, shrimp farming systems use ~57 m^3 ^kg^−1^ water shrimp [13, 19]. The effects of having excessive discharges of effluent from shrimp farms include organic enrichment of the sediment and water, hypernutrification and discharge of high concentrations of heterotrophic bacteria, nitrifying, and types of vibrio [20]; such alterations influence the distribution and abundance of benthic species [21]. In the northwest of Mexico, the recovery of N is 25 to 35%, and discharge is from 27 to 35% with water exchange rates that can exceed 16% daily [9, 19]. Our hypothesis is that farming systems can reduce turnover rates and leverage the recycling of nutrients in order to promote ecoefficiency in shrimp farming. The study was conducted on a farm in semi-intensive shrimp farming designed with interconnected ponds (unique to Mexico) for reuse; water exchange rates <2% water. The goal was to evaluate the effect of this interconnected pond design with low water exchange rates on water quality, production parameters, material flows, and nitrogen contribution to the environment.

## 2. Materials and Methods

### 2.1. Area of Study

**Figure 1:**
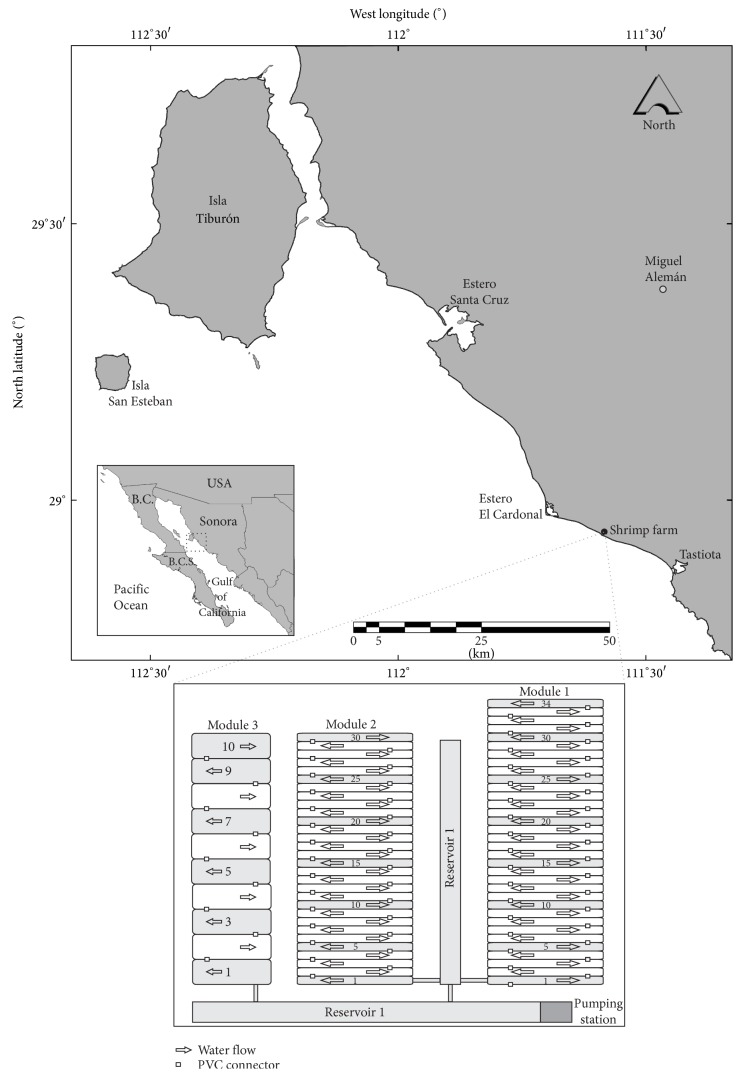
Study shrimp farm location and design of three modules with interconnected ponds. The arrows show the flow of water between the ponds. Studied ponds are designated with numbers.

Shrimp aquaculture farm Acuícola Polo, S.A. de C.V., is located in northwest Mexico (Figure 1). The farm consists of three modules; Module 1 (M1) has 34 rectangular earthen ponds 1 ha. each (average depth 1.2 m, volume of 12,000 m^3^), and Module 2 (M2) has 30 ponds of the same depth and volume as that of the M1, and Module 3 (M3) has 10 ponds of three ha. each (average depth of 1.5 m and volume of 46,500 m^3^).

The water was pumped directly from an inlet open to the sea and channeled to two reservoirs (reservoir 1 and reservoir 2). From these channels reservoirs, water flowed from the first to the last tank by plastic tubes (Figure 1). The tanks of each module were maintained interconnected by plastic tubes of 1 m diameter placed along the edge perimeters of the ponds (Figure 1). In each module the water flowed through the first pond and then flowed into the other and so forth until reaching the last pond. This design allowed foe water reuse from the first pond to the last throughout the crop cycle (Figure 1).

Water exchange rates were performed daily with a percentage of 1.6 ± 0.24% for modules 1 and 2 and 1.5 ± 0.22% for the M3. The estimates of water exchange rates were determined following the criteria of Wheaton [22].

In M1, M2, and M3 postlarvae of* L. vannamei* (PL_14_, average weight 1.1 mg) were seeded at densities of 30, 30, and 35 PL/m^−2^. The days of culture in both modules were 187 and 157 days. During this period the shrimp were fed daily three times a day (08:00, 14:00, and 20:00), with commercial feed (35% crude protein, 88% dry matter, and 8% lipids). The daily ration was estimated according to [9, 23]. During cultivation no fertilizer was added to the ponds.

### 2.2. Water Quality

The water quality parameters were monitored in the pumping station (a), reservoirs (b), and the water outlet for each of the 20 ponds studied (c) in the three modules (Figure 1).

At each sampling site temperature was recorded daily, as well as dissolved oxygen (DO) and salinity with YSI multisensor (Model YSI 85, YSI Incorporated, Yellow Springs, Ohio 45387 USA) and pH with a potentiometer Model Hanna 220A. Each week water transparency was recorded with a Secchi disk. Every two weeks, water samples were collected in 1 L plastic bottles to determine suspended solids (inorganic and organic) nutrients, chlorophyll *a*. Water samples were kept on ice during transport to the laboratory.

### 2.3. Total Suspended Solids, Particulate Organic Matter, and Chlorophyll *a*

The water samples were filtered through a vacuum pump through glass fiber filters Whatman GF/C of 47 mm diameter and 1.4 *μ* pore opening. To determine suspended solids and organic matter the Strickland and Parsons technique [24] was carried out. Chlorophyll *a* was determined with the procedure of Parsons et al. [25] using 90% acetone for removal of pigments.

### 2.4. Dissolved Inorganic Nutrients

Previously filtered water was used to determine the concentration of dissolved inorganic nutrients (NO_2_-N, NO_3_-N, and NH_4_-N) by a spectrophotometer Hach DR/5000 using methods of diazotization with ferrous sulfate acid medium (Method 8507) for NO_2_^−^-N, cadmium reduction to NO_2_^−^-N, and diazotization (Method 8171) for NO_3_^−^-N and salicylate (Method 8155) for NH_4_^+^-N following the procedure described in the manual [26].

### 2.5. Total Nitrogen by Kjeldahl Method

Water samples collected were processed in triplicate following the micro Kjeldahl method that included digestion with sulfuric acid and hydrogen peroxide, according to the method 8075 of procedures spectrophotometer manual [26].

### 2.6. Chemical Flows and Partial Mass Balance Calculation

The estimate of the daily nutrient water quality was obtained with weekly data interpolation [19]. The concentrations were multiplied by the daily water exchange to determine the total weight of each parameter in the exchanged water. Similarly, the mass flow of each parameter which entered and exchanged through the ponds was calculated based on water entering from the harbor. The net water balance (kg ha^−1^) was estimated from the difference between the inputs and outputs [19].

Farm records were used to quantify the amount of food added to each pond. The concentration of nitrogen in the feed used and shrimp harvested was calculated according to [16, 19].

To estimate the nitrogen content in the associated macrofauna we used the average value reported in studies of [16, 27–29].

The volume of refilled water is based on the records of the farm. Evaporation and precipitation were estimated based on the records of the weather station of the National Water Commission for the Costa de Hermosillo Sonora Mexico [30].

Water flows were calculated based on volumes of water exchange rate, evaporation, and precipitation. The inputs of nutrients via atmospheric precipitation and nitrification and fixation of nutrients by microalgae were not considered for this study. Estimates of flows admission and release of N were expressed in kg ha^−1^ cycle^−1^.

### 2.7. Statistical Analysis

Tests for homoscedasticity and normality were applied to determine the use of parametric or nonparametric methods [30, 31]. ANOVA Kruskal-Wallis was used to determine differences between the levels of the variables of water and they were used to evaluate production parameters of the three modules studied. In cases in which there were significant differences, multiple comparisons tests were run. In all cases the level of significance was 0.05. The data were processed using the Number Cruncher Statistical System software [32].

## 3. Results and Discussion

In this culture model with low turnover and reuse of water, shrimp growth was not limited by the quality of water variable, keeping water quality within safe levels [33, 34]. The averages of the variables of water during the growing season are presented in Table 1. Concentrations of DO in a few of the weeks in the mornings were below recommended levels (<2 mg L^−1^), this is because no mechanical aeration was used in addition to the combined effect of high natural productivity, temperature, and salinity prevailing in the water during this period. The average dissolved oxygen varied from 2.8 mg L^−1^ (morning) to 6.3 mg L^−1^ (afternoon). Some studies show that values < 2 mg L^−1^ of DO can be critical for the growth of shrimp [35, 36], but in our study no mortalities were observed. Comparatively, M1 and M2 had similar water quality conditions, while M3 had higher water temperature since cultivation began a month later, the DO was lower and the NH_4_-N was the highest and this is mainly attributed to the higher planting density (35 PL m^−2^).

Lower salinity values (37.9 psu) were recorded at beginning of cultivation, while at the end values reached 45 psu. However, the low rate of water exchange salinity had a small increase (9 psu) and remained at levels comparable to other studies in shrimp farms in Northwestern Mexico: 42 to 48 psu [19], 45 ± 5 psu [13], and 41 to 42 psu [37].

Production results are presented in Table 2. The survival rate varied between 70.9 and 78.0% with an average weight that was between 17 and 20 g, with no significant difference between the modules. Shrimp production for M1, M2, and M3 was 4,285, 4,250, and 4,683 kg ha^−1^, respectively (Table 2).

The concentrations of nitrogen compounds (NH_4_-N and NO_2_-N, NO_3_-N) remained at comparable levels to those seen in a traditional semi-intensive culture of* L. vannamei* in Northwestern Mexico, where Casillas-Hernández et al. [34] reported 0.1 to 0.1 mg L^−1^ of NH_4_-N and 0.05 mg L^−1^ of NO_2_-N 0.5 mg L^−1^ of NO_3_-N while Miranda et al. [13] reported 0.1 mg L^−1^ of NH_4_-N 0.04 mg L^−1^ NO_2_-N and 0.1 mg L^−1^ of NO_3_-N. The concentrations of NO_3_-N observed in this study >1 mg L^−1^ are consistent with previous studies on farms in the regions ~2 mg L^−1^ [38] and ~3 mg L^−1^ [37]. These levels of NO_3_-N indicate an efficient nitrification within farming systems [39].

TNK concentration observed was similar to those reported by Miranda et al. [13]. ~2 mg L^−1^ suggested that the system of interconnected ponds has low water exchange rate but provided efficient remineralization of N. Dissolved inorganic nitrogen (DIN = NO_2_-N + NO_3_-N + NH_4_-N) maintained average concentrations >1 mg L^−1^ similar to that observed by Wang et al. [40] ~2 mg L^−1^ for intensive cultivation of* L. vannamei* (62–227 ind m^−2^) in ponds treated with probiotics. This indicates that the interconnected ponds can act as remineralization lagoons where the accumulation of N can promote the development of natural productivity.

The biomass of phytoplankton in the ponds was higher in the middle sections and at the end of the modules studied, indicating a higher level of eutrophication as water was being reused. Evidence of this was provided by the concentration of Chlorophyll *a* in our study which was higher than those reported for crops of traditional semi-intensive systems for* L. vannamei* in Mexico, which were 10 ± 8 mg m^−3^ [19] and 6 ± 3 mg m^−3^ [13] and 15 ± 1 to 17 ± 2 mg m^−3^ [34] and 8 ± 3 to 16 ± 2 mg m^−3^ [37].

Total suspended solids, from both inorganic and particulate organic matter showed similar concentrations between M1, M2, and M3 and are within a range comparable with that reported for semi-intensive culture of* L. vannamei* in Mexico TSS: 96 ± 5, SSI: 69 ± 36 and POM: 27 ± 7 mg L^−1^ [13], TSS: 124 ± 11 to 153 ± 12, and POM: 30 ± 3 to 38 ± 3 mg L^−1^ [34]. Our results showed that the culture system of interconnected ponds maintained a proper process of remineralization of organic matter which was provided by shrimp feces and leftover food, so nutrients helped keep significant concentrations of phytoplankton biomass, promoting the presence of natural food in the culture system.

One way to assess the efficiency of water is estimating the volume of water used to produce one kg of shrimp per crop cycle. In our study the efficiency was 5 to 6.5 m^3 ^kg^−1^ cycle^−1^ at 160 to 190 days of culture, which is significantly lower compared to other semi-intensive crops. Reference [41] estimated a worldwide range of 39 and 199 m^3 ^kg^−1^ cycle^−1^ for semi-intensive and intensive shrimp culture systems, respectively. Until recently values 100–200 m^3 ^kg^−1^ cycle^−1^ were considered to be efficient for semi-intensive systems [42]. In Northwest Mexico, semi-intensive systems have shown a broad range [38] and obtained an average of 45 m^3 ^kg^−1^ cycle^−1^ with 7% daily water exchange; Casillas-Hernández et al. [9] obtained 62–71 m^3 ^kg^−1^ cycle^−1^ with daily turnover of 11%; Miranda et al. [13] reported values of 101–105 m^3 ^kg^−1^ cycle^−1^ with a turnover of 13% day^−1^. Studies in shrimp cultures with low water exchange in Mexico have reported rates of 9 to 17 m^3 ^kg^−1^ cycle^−1^ with 3–5% daily turnover [19], 17 to 38 m^3 ^kg^−1^ cycle^−1^ with 5% daily turnover in 140-day cycles [15], and 17 to 21 m^3 ^kg^−1^ cycle^−1^ with 5% daily turnover in of 120-day cycles [37].

The feed conversion factor (FCF) obtained in the present study (Table 2) was lower than that reported (2.2) by Miranda et al. [13] and remained within the range (1.2 to 1.8) obtained by Páez-Osuna et al. [19] in farms in the Northwest of Mexico. The global average of FCA for semi-intensive shrimp farms is 1.8 [6, 15]. This indicates that the administration and feed efficiency in our study was similar to that obtained in traditional farms, but with more efficient use of water, thus improving overall efficiency since it promotes recycling of nutrients in ponds and increases primary productivity. This has been observed previously by [43] that it is feasible to reduce the food conversion factor.

The evaluated model of interconnected ponds with low water exchange was more efficient because it exported less volumes of TSS (660 to 1,566 kg ha^−1^), ISS (441 to 1,280 kg ha^−1^), POM (221 to 407 kg ha^−1^), TON: total organic nitrogen (12–36 kg ha^−1^), and TIN: total inorganic nitrogen (8–15 kg ha^−1^) compared to other reports from semi-intensive farms in Mexico that operate with traditional ponds; TSS: 12,696 to 17,539, POM: 3,054 to 5,349, and TIN: 18.6 to 20.8 kg ha^−1^ [34]; TSS: 8,479, ISS: 7,562, POM 917, TON: 103, and TIN: 19 kg ha^−1^ [13]. In our study net contributions of materials are similar to that observed in cultures operated with lower stocking densities (14 to 20 ind m^−2^) and turnover rates of 3 to 5%, TSS: 1591 and POM: 199 kg ha^−1^ [19].

Net discharges of the materials per kg of shrimp produced (TSS: 0.16 to 0.37, ISS: 0.1 to 0.3, MOP: 0.1, TON: 0003 to 0008, TIN: 0.002 to 0003, and chlorophyll *a*: 0.0001 to 0.0003 kg^−1^ shrimp) also found that the tested model has better efficiency than traditional semi-intensive crops TSS: 4.2, ISS: 3.8, POM: 0.5, TON: 0.1, TIN: 0.01, and chlorophyll *a*: 0.0005 kg^−1^ shrimp [13] and TSS: 4.3 to 5.3, POM: 0.9 to 1.8, TIN: 0.006, and chlorophyll *a*: 0.001 kg^−1^ shrimp [34].

Table 3 presented the N mass balance calculations for each of the modules. In each case the most important source of N to the system was from the artificial food in M1 (82%), M2 (83%), and M3 (84%). Organic N input from water for M1 accounted for 12% and for M2 and for M3 10%. The inorganic N for M1, M2, and M3 was 6%. The N content in postlarvae was almost negligible (<0.1% in all the three modules).

**Figure 2:**
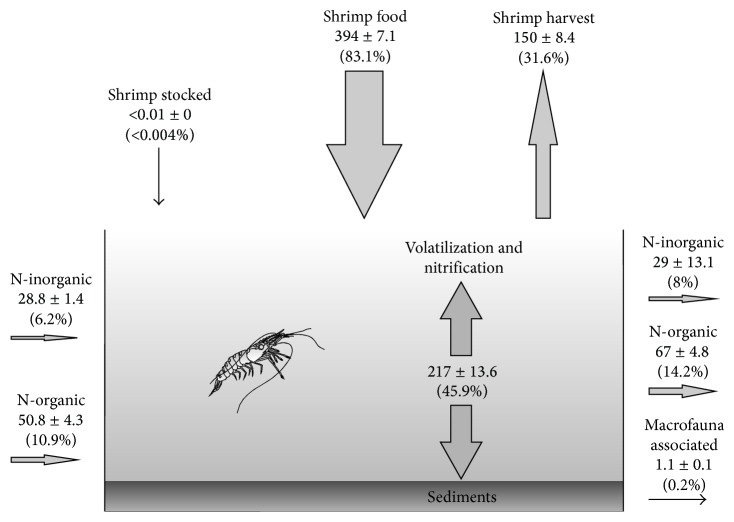
Mass balance for nitrogen in shrimp farm with interconnected ponds. Units in kg ha^−1^ cycle^−1^ (±SD) and parenthesis; values represent mean percentage of variables.

According to the mass balance results the greatest loss of N was via sedimentation and volatilization of ammonium; values in the modules M1, M2, and M3 were 47%, 48%, and 43%, respectively. The amount of N removed during harvest shrimp in M1, M2, and M3 was 31%, 30%, and 34%, respectively. The discharge of effluent via organic N for M1, M2, and M3 represented 14% and 15%. The inorganic N accounted for and was 8% in all modules. The amount of N removed by the associated macrofauna was <0.3% in all ponds. The various inflows and outflows of N are presented in Table 3. The mass balance results indicated that the supplied food was the main N input source to the system (82–84%) (Figure 2). This coincides with previous reports for intensive and semi-intensive systems where food can contribute between 71 and 97% of total N [19, 34, 36, 44–46]. With regard to sources of N discharge, the other studies mentioned above are consistent with those observed in our study, where the main forms of N are found in the sedimentation and are volatilized in the form of ammonia. The N retrieved vis-à-vis biomass harvested shrimp was 30 to 34%, which suggests better usage of N in the food provided. Other traditional semi-intensive shrimp farms in the Northwest of Mexico reported values of 20–24% [47]. In other countries values of N vary from 18 to 27% [12, 16–18]. Based on our results, the modular design of interconnected ponds with low turnover and reuse of water significantly improves the recovery of N as shrimp tissue (Figure 2). The N sedimented and volatilized were not quantified separately; however it is possible that most of the N that had been deposited in the pond sediment is in the form of organic nitrogen sequestered in organic matter, considering that in this design the flow of water is very low favoring sedimentation in the ponds. It is assumed that the organic N in sediment was the most abundant form since sedimentation of organic matter was caused by the sum of accumulated leachated commercial feed and shrimp feces as suggested [11, 36]. Ammonia volatilization is not considered a significant loss in ponds when ammonia levels are <1 mg L^−1^ and pH 7.5–8.5 [48–50] as observed in our study. In addition, [12] mentions that the wind or mechanical ventilation are other factors that influence the presence and volatilization of ammonia (NH_3_-N). Our results showed that the dominant species and chemistry in the aquaculture system was NH_4_-N. This indicates a low loss by volatilization because ponds were not aerated. The organic form of N in the water was the most abundant, which coincides with Jackson et al. [12] where they reported a close relationship between chlorophyll *a* and particulate organic N, assuming that most of POM is due to the presence of phytoplankton.

Modular design in low turnover and high water retention time allowed complete nitrification. This is reflected by elevated levels of NO_3_-N. The levels of organic N were similar to what was reported (~2 mg L^−1^) by [13] although in their study turnover rate was 12% day^−1^. In our aquaculture system with low water exchange, the low levels of NH_4_-N indicate efficient nitrification, but these conditions are difficult to achieve in shrimp farms with high water exchange rate [13].

In Table 4 the nutrient flows and material discharge via water is presented. The greatest discharges corresponded to TSS (660 to 1,566 kg ha^−1^); the ISS varied from 441 to 1,280 kg ha^−1^ and MOP 221 to 407 kg ha^−1^. Chlorophyll *a* values varied from 0.50 to 1.27 kg ha^−1^. The contribution from nitrogen compounds was dominated by TNK with interval of 12 to 36 kg ha^−1^, followed by NO_3_-N 6.75 to 14.8 kg ha^−1^, NH_4_-N 0.35 to 0.92 kg ha^−1^, and NO_2_-N 0.03 to 0.08 kg ha^−1^. In all three modules the organic N (TNK) exceeded inorganic N levels. In our study, the estimated net N contribution to the environment was 24 kg ton^−1^ at shrimp planting densities of 30–35 ind m^−2^. Whether N loss in semi-intensive culture with* L. vannamei* is variable depending on planting density (ds), the rate of water exchange (tr), and days in culture (dc), for example, 18 kg N, ds: 11 ind m^−2^, tr: 4.7%, and dc: 95–162 days [51]; 29 kg N, ds: 17 ind m^−2^, tr: 3–5%, and dc: 95–165 days [19]; and 72 kg N, ds: 15 ind m^−2^, tr: 11%, and dc: 203 days [9]. The levels obtained in this study were only surpassed by the study in [51] but with a considerably lower density. Therefore the system of interconnected ponds with low turnover had a lower environmental N loss. Environmental losses of N in intensive shrimp farming of* L. vannamei* vary between of 38–44 kg N ton^−1^ [44], 53 kg N ton^−1^ [52], and 72 kg N ton^−1^ [12]. This provides evidence that cropping systems with reduced or no turnover rate can help reduce significantly N discharge to the environment and its productions are comparable to those systems that handle high turnover rates.

Our study provides evidence that by interconnecting ponds nutrient recycling is favored. Construction engineering with interconnected ponds promotes the growth of primary producers such as pond microalgae [20], which produce sugars, proteins, and other components required by shrimp for various biochemical processes such as respiration, digestion, and biosynthesis, as well as the energy required for movement and nutrition [53]. This has a practical benefit because it can improve the conversion factor of artificial food for shrimp biomass.

The best recycling of nutrients and the promotion of microalgae also favor the development of heterotrophic microorganisms that feed primarily on organic matter in the culture ponds [54]. This web-established food in the ponds made nutrient recycling more efficient [55], with additional practical benefits. Hence, with this water quality cropping system, nutrition and health status of the shrimp are improved [54, 56].

As previously noted in this study, the system of ponds interconnected with low turnover rates significantly increases the reuse and efficiency in water use providing economic benefits (cost savings of retail electricity and water booster factor reduction FCR) and environmental benefits (healthier aquaculture systems and crop effluent with lower contribution of important nutrients and organic matter).

We believe that the interconnection of ponds is a production model technically feasible and is compatible with other biotech innovations, for example, the implementation of bioreactors in cropping systems to facilitate the growth of beneficial bacteria consortia. In short, the study results provide elements to reduce production costs of systems of semi-intensive shrimp farming in Mexico, while also reducing environmental impacts.

In a recent review [57], it is mentioned that aquaculture must have the best practices of cultivation and the ecosystem approach to better integrate aquaculture in inland basins and coastal areas with more efficient use of land and water.

## 4. Conclusions

Our study provides evidence that by interconnecting ponds with low water exchange then nutrient recycling is favored and promotes growth of the food web with organisms working in nutrition and semi-intensive production of* Litopenaeus vannamei*.

According to the mass balance and flow of nutrients this culture system reduces the flow of solid, particulate organic matter, and nitrogen compounds into the environment and significantly increases the efficiency of water, when compared with a traditional culture system.

With this culture system, it is possible to recover up to 31.6% of the total nitrogen entering the pond and produce more than 4,000 kg ha^−1^ of shrimp.

The production system of interconnected ponds is technically feasible, and it also can incorporate innovations such as the use of bioreactors to increase consortia of heterotrophic microorganisms and other beneficial bacteria that help to improve the ecoefficiency of shrimp farming.

## Figures and Tables

**Table 1:** Mean value (±SD) of water quality variables in three modules during a 157–187-day trial.

Water quality variable	M1	M2	M3	*P *
Temperature (6 h°C)	28.3 ± 2.59^a^	28.1 ± 2.52^a^	29.3 ± 2.0^b^	<0.001^*∗*^
Temperature (14 h°C)	30.7 ± 2.14^a^	30.9 ± 2.18^a^	31.3 ± 2.08^b^	<0.001^*∗*^
DO. (6 h mg L^−1^)	3.57 ± 1.53^b^	3.58 ± 1.43^b^	2.81 ± 1.73^a^	<0.001^*∗*^
DO. (14 h mg L^−1^)	5.45 ± 1.52^b^	6.37 ± 1.0^c^	4.72 ± 1.65^a^	<0.001^*∗*^
Salinity (‰)	38.7 ± 3.24^a^	38.5 ± 3.0^a^	40.4 ± 5.53^a^	0.28
pH (14 h)	8.22 ± 0.22^a^	8.2 ± 0.21^a^	8.19 ± 0.28^a^	0.40
Transparency (14 h cm)	38.5 ± 13.5^a^	39.7 ± 9.8^a^	46.6 ± 19.1^b^	<0.001^*∗*^
TSS (mg L^−1^)	128.4 ± 49.7^a^	126.2 ± 54.7^a^	173.0 ± 52.0^a^	0.31
ISS (mg L^−1^)	108.2 ± 45.1^a^	106.5 ± 48.8^a^	116.6 ± 45.9^a^	0.24
POM (mg L^−1^)	20.4 ± 8.4^a^	19.9 ± 8.0^a^	20.4 ± 8.8^a^	0.89
Chlorophyll *a* (mg m^−3^)	32.4 ± 15.8^ab^	38.4 ± 20.0^b^	29.1 ± 21.1^a^	0.02^*∗*^
NO_2_-N (mg L^−1^)	0.0043 ± 0.0021^a^	0.0043 ± 0.0024^a^	0.0062 ± 0.0058^a^	0.06
NO_3_-N (mg L^−1^)	1.4 ± 0.57^a^	1.3 ± 0.49^a^	1.37 ± 0.37^a^	0.40
NH_4_-N (mg L^−1^)	0.08 ± 0.05^a^	0.07 ± 0.05^a^	0.11 ± 0.05^b^	<0.001^*∗*^
TNK (mg L^−1^)	2.5 ± 1.3^ab^	2.14 ± 1.1^a^	2.8 ± 1.11^bc^	<0.001^*∗*^

Different letters among modules for each variable indicate significant differences, ^*∗*^indicate probablity: ANOVA Kruskal-Wallis, and *P* < 0.05.

**Table 2:** Water exchange, survival, final body weight, total production, and feed conversion ratio per module during trial *Litopenaeus vannamei*.

Variables	M1	M2	M3
Water exchange day (%)	1.6 ± 0.24	1.6 ± 0.24	1.5 ± 0.22
Survival (%)	74.8 ± 7.6^a^	70.9 ± 4.7^a^	78.0 ± 6.7^a^
Day of trial	187	187	157
Stocking density (PL/m^2^)	30	30	35
Water flow (m^3^ ha^−1^ cycle^−1^)	27,700	27,700	24,100
Final body weight (g)	19.14 ± 0.69^b^	20.0 ± 0.82^b^	17.17 ± 0.88^a^
Production (Kg ha^−1^)	4285 ± 292^a^	4250 ± 202^a^	4683 ± 384^b^
Feed added (Kg ha^−1^ cycle^−1^)	7801 ± 282	8074 ± 242	8115 ± 495
Feed conversion ratio	1.82 ± 0.11^a^	1.9 ± 0.04^a^	1.74 ± 0.18^a^

Different letters among modules for each variable indicate significant differences (ANOVA Kruskal-Wallis, one via *P* < 0.05).

**Table 3:** Partial nutrient budget N, for different modules during a 157–187-day trial.

Variables	(kg ha^−1^ cycle^−1^)	(%)
*Module 1 *		
Feed shrimp	384.43	81.78
Postlarval shrimp	0.02	<0.01
N-inorganic	28.86	6.14
N-organic (TKN)	56.79	12.08
Total input	470.10	100.0
Macrofauna	1.1926	0.2537
Biomass shrimp	146.09	31.07
N-inorganic	39.96	8.50
N-organic (TKN)	63.76	13.56
Sedimentation and volatilization	219.09	46.61
Total output	470.10	100.0

*Module 2 *		
Feed shrimp	397.88	83.43
Postlarval shrimp	0.01987	0.004
N-inorganic	30.55	6.41
N-organic (TKN)	48.46	10.16
Total input	476.92	100.0
Macrofauna	0.9228	0.1935
Biomass shrimp	144.87	30.37
N-inorganic	35.53	7.45
N-organic (TKN)	65.35	13.70
Sedimentation and volatilization	230.24	48.28
Total output	476.92	100.0

*Module 3 *		
Feed shrimp	399.92	84.33
Postlarval shrimp	0.01987	0.004
N-inorganic	27.13	5.72
N-organic (TKN)	47.14	9.94
Total input	474.22	100.0
Macrofauna	1.2483	0.1935
Biomass shrimp	159.62	33.66
N-inorganic	37.53	7.91
N-organic (TKN)	72.73	15.34
Sedimentation and volatilization	203.09	42.83
Total output	474.22	100.0

**Table 4:** Fluxes estimated (kg ha^−1^) (mean ± standard error) of incorporated, discharged, and net loading material (outlet − inlet) via water for shrimp culture in three modules.

Variables	M1			M2			M3		
	Inlet (kg ha^−1^)	Outlet (kg ha^−1^)	Net load (kg ha^−1^)	Inlet (kg ha^−1^)	Outlet (kg ha^−1^)	Net load (kg ha^−1^)	Inlet (kg ha^−1^)	Outlet (kg ha^−1^)	Net load (kg ha^−1^)
NH_4_^+^-N	1.91	2.26	0.35	1.48	2.40	0.92	2.26	2.75	0.49
NO_2_^−^-N	0.08	0.12	0.04	0.08	0.11	0.03	0.11	0.19	0.08
NO_3_^−^-N	26.9	40.7	13.7	29.0	35.7	6.75	29.2	44.1	14.8
TKN	56.7	68.7	11.9	48.4	70.4	21.9	54.8	91.1	36.3
TSS	2750.3	4316.5	1566.2	2944.7	3605.3	660.5	3045.3	4556.6	1511.3
ISS	2407.3	3688.1	1280.7	2556.8	2998.1	441.3	2669.1	3784.3	1115.1
POM	342.9	660.0	317.0	387.9	608.9	221.0	376.1	783.9	407.7
CL *a *	0.50	1.00	0.50	0.55	1.19	0.64	0.35	1.62	1.27

## References

[1] Troell M., Halling C., Neori A. (2003). Integrated mariculture: asking the right questions. *Aquaculture*.

[2] Rivera-Monroy V. H., Torres L. A., Bahamon N., Newmark F., Twilley R. R. (1999). The potential use of mangrove forests as nitrogen sinks of shrimp aquaculture pond effluents: the role of denitrification. *Journal of the World Aquaculture Society*.

[3] Mao Y., Yang H., Zhou Y., Ye N., Fang J. (2009). Potential of the seaweed *Gracilaria lemaneiformis* for integrated multi-trophic aquaculture with scallop *Chlamys farreri* in North China. *Journal of Applied Phycology*.

[4] Miranda-Baeza A., Voltolina D., Frías-Espericueta M. G., Izaguirre-Fierro G., Rivas-Vega M. E. (2009). Budget and discharges of nutrients to the Gulf of California of a semi-intensive shrimp farm (NW Mexico). *Hidrobiológica*.

[5] Lezama-Cervantes C., Paniagua-Michel J., Zamora-Castro J. (2010). Utilizando tapetes microbianos en un sistema de recirculación. *Latin American Journal of Aquatic Research*.

[6] Martinez-Cordova L. R., Campaña-Torres A., Porchas-Cornejo M. A. (2002). Promotion and contribution of biota in low water exchange ponds farming blue shrimp *Litopenaeus stylirostris* (Stimpson). *Aquaculture Research*.

[7] Lawrence A. L., Castille F., Velasco M., Samocha T., Browdy C. L., Jory D. E. (2001). ‘Enviromentally friendly’ or ‘least polluting’ feed management program for shrimp farming. *The New Wave. Proceedings of the Special Session on Sustainable Shrimp Culture*.

[8] García-Sanz T., Ruiz J. M., Pérez M., Ruiz M. (2011). Assessment of dissolved nutrients dispersal derived from offshore fish-farm using nitrogen stable isotope ratios (*δ*
^15^N) in macroalgal bioassays. *Estuarine, Coastal and Shelf Science*.

[9] Casillas-Hernández R., Magallón-Barajas F., Portillo-Clarck G., Páez-Osuna F. (2006). Nutrient mass balances in semi-intensive shrimp ponds from Sonora, México using two feeding strategies: trays and mechanical dispersal. *Aquaculture*.

[10] COSAES Informe final sanidad e inocuidad camarón 2014. http://www.cosaes.com/.

[11] Burford M. A., Williams K. C. (2001). The fate of nitrogenous waste from shrimp feeding. *Aquaculture*.

[12] Jackson C., Preston N., Thompson P. J., Burford M. (2003). Nitrogen budget and effluent nitrogen components at an intensive shrimp farm. *Aquaculture*.

[13] Miranda A., Voltolina D., Brambilla-Gámez M. A., Frías-Espericueta M. G., Simental J. (2007). Effluent characteristics and nutrient loading of a semi-intensive shrimp farm in NW Mexico. *Life and Environment*.

[14] Kittiwanich J., Songsangjinda P., Yamamoto T., Fukami K., Muangyao P. (2012). Modeling the effect of nitrogen input from feed on the nitrogen dynamics in an enclosed intensive culture pond of black tiger shrimp (*Penaeus monodon*). *Coastal Marine Science*.

[15] Martínez-Córdova L. R., López-Elías J. A., Leyva-Miranda G., Armenta-Ayón L., Martínez-Porchas M. (2011). Bioremediation and reuse of shrimp aquaculture effluents to farm whiteleg shrimp, *Litopenaeus vannamei*: a first approach. *Aquaculture Research*.

[16] Boyd C. E., Teichert-Coddington D. (1995). Dry matter, ash, and elemental composition of pond-cultured *Penaeus vannamei* and *Penaeus stylirostris*. *Journal of the World Aquaculture Society*.

[17] Funge-Smith S. J., Briggs M. R. P. (1998). Nutrient budgets in intensive shrimp ponds: implications for sustainability. *Aquaculture*.

[18] Burford M. A., Preston N. P., Glibert P. M., Dennison W. C. (2002). Tracing the fate of ^15^N-enriched feed in an intensive shrimp system. *Aquaculture*.

[19] Páez-Osuna F., Guerrero-Galván S. R., Ruiz-Fernández A. C., Espinoza-Angulo R. (1997). Fluxes and mass balances of nutrients in a semi-intensive shrimp farm in north-western Mexico. *Marine Pollution Bulletin*.

[20] Barraza-Guardado R. H., Arreola-Lizárraga J. A., López-Torres M. A. (2013). Effluents of shrimp farms and its influence on the coastal ecosystems of bahía de Kino, Mexico. *The Scientific World Journal*.

[21] Barraza-Guardado R. H., Martínez-Córdova L. R., Enríquez-Ocaña L. F., Martínez-Porchas M., Miranda-Baeza A., Porchas-Cornejo M. A. (2014). Effect of shrimp farm effluent on water and sediment quality parameters off the coast of Sonora, Mexico. *Ciencias Marinas*.

[22] Wheaton F. W. (1982). *Acuacultura: Diseño y Construcción de Sistemas*.

[23] Seiffert Q. W., Foes G. K. Manejo alimentar através de bandejas de alimentacao.

[24] Strickland J. D. H., Parsons T. R. (1972). *A Practical Handbook of Seawater Analysis*.

[25] Parsons T. R., Maita Y., Lalli C. M. (1984). *A Manual Chemical and Biological Methods for Seawater Analysis*.

[26] Hach (2005). Nitrogen, total Kjeldahl Nessler method. *Journal of Association of Official Analytical Chemists*.

[27] Anger K., Harms J. (1990). Elemental (CHN) and proximate biochemical composition and decapod crustacean larvae. *Comparative Biochemistry and Physiology B*.

[28] Tacon A. G. T. (1990). *Standard Methods for the Nutrition of Farmed Fish and Shrimp. Vol 2. Nutrient Sources and Composition*.

[29] Zafar M. S., Huda M., Abdul H. M. (2004). Biochemical composition in *Scilla serrata* (Foskal) of chakaria Sundarban area. Bangladesh. *Pakistan Journal of Biological Sciences*.

[30] Sokal R., Rohlf F. (2000). *Biometry*.

[31] Zar J. (1999). *Biostatistical Analysis*.

[32] NCSS (2007). *Number Cruncher Statistical System: User Guide*.

[33] Villalón J. R. (1991). Practical manual for semi-intensive commercial production of marine shrimp. *Sea Grant College Program*.

[34] Casillas-Hernández R., Nolasco-Soria H., García-Galano T., Carrillo-Farnes O., Páez-Osuna F. (2007). Water quality, chemical fluxes and production in semi-intensive Pacific white shrimp (*Litopenaeus vannamei*) culture ponds utilizing two different feeding strategies. *Aquacultural Engineering*.

[35] Allan G. L., Maguire G. B. (1991). Lethal levels of low dissolved oxygen and effects of short-term oxygen stress on subsequent growth of juvenile *Penaeus monodon*. *Aquaculture*.

[36] Lemonnier H., Faninoz S. (2006). Effect of water exchange on effluent and sediment characteristics and on partial nitrogen budget in semi-intensive shrimp ponds in New Caledonia. *Aquaculture Research*.

[37] Porchas-Cornejo M. A., Martínez-Córdova L. R., Ramos-Trujillo L., Hernández-López J., Martínez-Porchas M., Mendoza-Cano F. (2011). Effect of promoted natural feed on the production, nutritional, and immunological parameters of *Litopenaeus vannamei* (Boone, 1931) semi-intensively farmed. *Aquaculture Nutrition*.

[38] Hernández-Ibarra A. (1999). *Comportamiento de la calidad de agua en una granja camaronícola del noroeste de México [Tesis de Licenciatura]*.

[39] Timmons M. B., Ebeling J. M., Weaton F. W., Summerfelt S. T., Vinci B. J. (2002). *Recirculating Aquaculture Systems*.

[40] Wang Y.-B., Xu Z.-R., Xia M.-S. (2005). The effectiveness of commercial probiotics in northern white shrimp *Penaeus vannamei* ponds. *Fisheries Science*.

[41] Hopkins J. S., Villalón J., Wyban J. A. Synopsis of industrial panel input on shrimp pond management.

[42] Clifford H. C., Wyban J. (1992). Marine shrimp pond management: a review. *Proceedings of the Special Session on Shrimp Farming*.

[43] Otoshi C. A., Montgomery A. D., Matsuda E. M., Moss S. M. (2006). Effects of artificial substrate and water source on growth of juvenile Pacific white shrimp, *Litopenaeus vannamei*. *Journal of the World Aquaculture Society*.

[44] Briggs M. R. P., Funge-Smith S. J. (1994). A nutrient budget of some intensive marine shrimp ponds in Thailand. *Aquaculture & Fisheries Management*.

[45] Thakur D. P., Lin C. K. (2003). Water quality and nutrient budget in closed shrimp (*Penaeus monodon*) culture systems. *Aquacultural Engineering*.

[46] Sahu B. C., Adhikari S., Dey L. (2013). Carbon, nitrogen and phosphorus budget in shrimp (*Penaeus monodon*) culture ponds in eastern India. *Aquaculture International*.

[47] Magallón-Barajas F. J., Arreola-Lizárraga J. A., Portillo-Clark G., Martínez-Córdova L. R. (2009). Capacidad de carga y capacidad ambiental en la camaronicultura. *Camaronicultura Sustentable*.

[48] Kochba M., Diab S., Avnimelech Y. (1994). Modeling of nitrogen transformation in intensively aerated fish ponds. *Aquaculture*.

[49] Hargreaves J. A. (1998). Nitrogen biogeochemistry of aquaculture ponds. *Aquaculture*.

[50] Lorenzen K., Struve J., Cowan V. J. (1997). Impact of farming intensity and water management on nitrogen dynamics in intensive pond culture: a mathematical model applied to Thai commercial shrimp farms. *Aquaculture Research*.

[51] Teichert-Coddington D. R., Martinez D., Ramirez E. (2000). Partial nutrient budgets for semi-intensive shrimp farms in Honduras. *Aquaculture*.

[52] Phillips K. Effects of used feed trays to feed adult Macrobrachium rosenbergrgii incommercial growoutponds.

[53] Ekasari J., Angela D., Waluyo S. H. (2014). The size of biofloc determines the nutritional composition and the nitrogen recovery by aquaculture animals. *Aquaculture*.

[54] Ray A. J., Dillon K. S., Lotz J. M. (2011). Water quality dynamics and shrimp (*Litopenaeus vannamei*) production in intensive, mesohaline culture systems with two levels of biofloc management. *Aquacultural Engineering*.

[55] Avnimelech Y. (2012). *Biofloc Technology. A Practical Guide Book*.

[56] Aguilera-Rivera D., Prieto-Davó A., Escalante K., Chávez C., Cuzon G., Gaxiola G. (2014). Probiotic effect of FLOC on Vibrios in the pacific white shrimp *Litopenaeus vannamei*. *Aquaculture*.

[57] Edwards P. (2015). Aquaculture environment interactions: past, present and likely future trends. *Aquaculture*.

